# The Effect of Two Different Intraoperative Doses of Dexmedetomidine on Postoperative Cognitive Dysfunction in Elderly Patients Undergoing Major Abdominal Surgery Under General Anesthesia: A Randomized Controlled Study

**DOI:** 10.7759/cureus.104579

**Published:** 2026-03-02

**Authors:** Debjit Ghosh, Mukesh Kumar, Mukesh Kumar, Shio Priye, Chandan Hessa

**Affiliations:** 1 Department of Anaesthesiology, Rajendra Institute of Medical Sciences (RIMS), Ranchi, IND; 2 Department of Cardiac Anaesthesia, Rajendra Institute of Medical Sciences (RIMS), Ranchi, IND; 3 Department of Superspeciality Anaesthesia, Rajendra Institute of Medical Sciences (RIMS), Ranchi, IND

**Keywords:** abdominal surgery, dexmedetomidine, elderly, general anaesthesia, moca, postoperative cognitive dysfunction

## Abstract

Background: Postoperative cognitive dysfunction (POCD) is a significant complication among elderly patients undergoing major abdominal surgery. Dexmedetomidine is commonly used as an adjunct to general anaesthesia, but the effect of different intraoperative dosing regimens on early postoperative cognitive outcomes remains unclear. This study evaluated the impact of two different dexmedetomidine doses on early postoperative cognitive outcomes.

Methods: A randomized, double-blind, controlled study was conducted on 72 elderly patients (≥60 years) undergoing elective major abdominal surgery under general anaesthesia. Patients were allocated into three groups (n = 24 each): Group P (placebo), Group D1 (dexmedetomidine 1 µg/kg bolus + 0.2 µg/kg/hr infusion), and Group D2 (dexmedetomidine 1.5 µg/kg bolus + 0.5 µg/kg/hr infusion). Cognitive function was assessed using the Montreal Cognitive Assessment (MoCA) scale preoperatively and at one, six, and 24 hours postoperatively. A decline of more than five points was considered significant POCD. Hemodynamic parameters and adverse events were also recorded.

Results: Baseline characteristics were comparable across all groups. At one hour postoperatively, MoCA scores were significantly higher in D1 and D2 compared with placebo (p = 0.046). At six hours, cognitive recovery was significantly better in D2, followed by D1, compared with placebo (p < 0.001). The incidence of significant cognitive decline (>5-point fall) was lowest in D1 and D2 (12.5% each) and highest in the placebo group (37.5%, p = 0.048). Dexmedetomidine groups showed more stable intraoperative hemodynamics without significant increases in adverse events.

Conclusion: Intraoperative dexmedetomidine significantly attenuates early POCD in elderly patients undergoing major abdominal surgery, with the higher dose demonstrating the greatest cognitive preservation. Dexmedetomidine may be safely incorporated into anaesthetic protocols to improve postoperative neurocognitive outcomes in high-risk elderly surgical patients.

## Introduction

Postoperative cognitive dysfunction (POCD) is one of the most common and clinically significant neurocognitive complications affecting elderly surgical patients. It manifests as impairments in memory, attention, executive function, language, and information processing, typically emerging within the early postoperative period and, in some cases, persisting for weeks or months. With the global increase in the elderly population undergoing major surgical procedures, POCD has become an important and growing public health concern. Previous studies have reported early postoperative cognitive impairment in 20-50% of elderly patients following major non-cardiac surgery, with approximately 10-15% experiencing persistent deficits several months after surgery. These cognitive impairments are associated with delayed recovery, prolonged hospitalization, increased morbidity, reduced functional independence, and diminished quality of life [1,2].

The pathophysiology of POCD is complex and multifactorial, involving systemic inflammatory responses, neuroinflammation, blood-brain barrier disruption, oxidative stress, and perioperative anesthetic and hemodynamic influences. Surgical trauma induces the release of inflammatory mediators such as interleukin-6 and tumor necrosis factor-α, which can activate central neuroinflammatory pathways and contribute to neuronal dysfunction and injury. Elderly patients are particularly susceptible to these effects because of age-related cerebral atrophy, reduced cognitive reserve, impaired cerebral autoregulation, and an exaggerated inflammatory response. This increased vulnerability underscores the need for perioperative strategies aimed at minimizing neurological insult and preserving cognitive function in older adults [3].

Dexmedetomidine, a highly selective α2-adrenergic receptor agonist, has gained increasing attention as a potential neuroprotective agent in the perioperative setting. It produces sedation that closely resembles physiological sleep, along with anxiolytic and analgesic effects, while causing minimal respiratory depression. Beyond its sedative properties, dexmedetomidine has demonstrated anti-inflammatory, anti-apoptotic, and sympatholytic effects that may attenuate mechanisms implicated in the development of POCD. Several clinical studies and meta-analyses have reported a reduction in early postoperative cognitive impairment and postoperative delirium among elderly patients receiving dexmedetomidine during surgery, across a range of surgical procedures [4,5].

Despite growing evidence supporting the neuroprotective role of dexmedetomidine, the optimal intraoperative dosing regimen for preventing POCD remains uncertain. Higher doses may offer enhanced anti-inflammatory and neuroprotective effects but are associated with an increased risk of bradycardia and hypotension, whereas lower doses may provide improved safety with potentially reduced cognitive benefit. Moreover, relatively few studies have directly evaluated dose-dependent cognitive effects of dexmedetomidine in elderly patients undergoing major abdominal surgery, a population at particularly high risk of early cognitive impairment due to extensive surgical stress, comorbidities, and prolonged anaesthesia exposure.

Given this background, the present study was designed to evaluate the effect of two different intraoperative dexmedetomidine dosing regimens on early postoperative cognitive function in elderly patients undergoing major abdominal surgery under general anaesthesia. The primary outcome was the change in Montreal Cognitive Assessment (MoCA) score from baseline at predefined early postoperative time points (one, six, and 24 hours after surgery). Secondary outcomes included the incidence of clinically significant postoperative cognitive decline, intraoperative hemodynamic parameters, and dexmedetomidine-related adverse events. The study further aimed to explore whether a dose-dependent difference exists between the two dexmedetomidine regimens in preserving early postoperative cognitive function while maintaining an acceptable safety profile.

## Materials and methods

Study design and population, intervention, comparison, and outcome (PICO) framework

This study was conducted as a prospective, randomized, double-blind, placebo-controlled trial at the Department of Anaesthesiology, Rajendra Institute of Medical Sciences (RIMS), Ranchi, Jharkhand, over a period of one and a half years. Ethical approval was obtained from the Institutional Research and Ethics Committee, and the trial was prospectively registered with the Clinical Trials Registry-India (CTRI No: CTRI/2024/08/072290).

The study methodology was structured according to the PICO framework as follows: Population (P): Elderly patients (≥60 years) undergoing elective major abdominal surgery under general anaesthesia; Intervention (I): Intraoperative dexmedetomidine administered in two different dosing regimens; Comparator (C): Placebo (0.9% normal saline infusion); Outcomes (O): Early postoperative cognitive function assessed by change in MoCA score at one, six, and 24 hours postoperatively; incidence of clinically significant POCD; intraoperative hemodynamic stability; and adverse events

Study population

Elderly patients aged 60 years and above scheduled for elective major abdominal surgery of expected duration two to four hours under general anaesthesia were screened for eligibility. Only patients classified as American Society of Anesthesiologists (ASA) physical status I or II were included.

Preoperative cognitive function was assessed using the MoCA scale. Patients with a baseline MoCA score ≥26 were included to exclude pre-existing cognitive impairment.

Patients were excluded if they had ASA physical status III or IV, refused consent, had known cognitive or psychiatric disorders (including dementia or schizophrenia), significant visual or hearing impairment, communication difficulties, or any condition likely to interfere with reliable cognitive assessment. Surgeries exceeding the defined duration window were excluded to ensure standardized timing of postoperative cognitive evaluation and to minimize circadian bias.

Sample size calculation

Sample size estimation was based on a clinically meaningful difference in postoperative cognitive scores, assuming an effect size (delta) of 0.70 and a standard deviation of 0.5. With a power of 80% and a two-sided alpha error of 5%, one-way analysis of variance (ANOVA) indicated a requirement of 72 patients, with 24 patients per group. The calculation was performed using STATA version 13 (StataCorp, College Station, TX, USA).

Randomization, allocation concealment, and blinding

Randomization was performed using a computer-generated random number sequence. Allocation concealment was ensured through sequentially numbered, sealed, opaque envelopes prepared by an independent investigator not involved in patient management or outcome assessment. Envelopes were opened only after patient enrollment and immediately before preparation of the study drug.

The study followed a double-blind design. Patients and the investigator responsible for postoperative cognitive assessment were blinded to group allocation. Study drugs were prepared by an anaesthesiologist not involved in intraoperative anaesthetic management or postoperative evaluation, ensuring maintenance of blinding throughout the study.

Intervention protocol

Participants were randomly allocated into three groups: Group P (Placebo): Received 0.9% normal saline infusion; Group D1: Received dexmedetomidine 1 μg/kg as a bolus over 10 minutes after induction, followed by a continuous infusion at 0.2 μg/kg/hour; and Group D2: Received dexmedetomidine 1.5 μg/kg as a bolus over 10 minutes after induction, followed by a continuous infusion at 0.5 μg/kg/hour. All infusions were discontinued at the time of skin closure.

Anaesthetic management

All patients underwent standardized pre-anaesthetic evaluation. In the operating room, standard monitoring including electrocardiography, non-invasive blood pressure, pulse oximetry, and capnography was instituted. Preoxygenation was performed for three minutes with 100% oxygen.

Anaesthesia was induced with fentanyl 2 μg/kg and propofol 1-2 mg/kg administered incrementally. Endotracheal intubation was facilitated using succinylcholine 1-1.5 mg/kg. Anaesthesia was maintained using oxygen, nitrous oxide, and sevoflurane, along with the allocated study infusion. Muscle relaxation was achieved with intermittent doses of vecuronium.

Depth of anaesthesia, intraoperative fluid therapy, and ventilatory parameters were managed according to standard institutional protocols and maintained consistently across all study groups. Neuromuscular blockade was reversed with neostigmine and glycopyrrolate, followed by extubation once standard recovery criteria were met.

Postoperative analgesia and sedation

Intraoperative opioid administration was standardized with fentanyl at induction. Postoperative pain was assessed using the Visual Analogue Scale (VAS) [6]. Rescue analgesia was administered according to a predefined institutional protocol when VAS scores reached ≥5.

No postoperative sedative medications or sedative infusions were administered before completion of postoperative cognitive assessments, ensuring that MoCA evaluation was not confounded by residual sedation.

Outcome assessment

Postoperative cognitive assessment was conducted by a blinded investigator using the MoCA scale at one hour, six hours, and 24 hours after discontinuation of anaesthesia and extubation, defining the early postoperative period [7].

A decrease of more than five points from baseline MoCA score was defined as clinically significant POCD. Intraoperative hemodynamic parameters, including heart rate and systolic and diastolic blood pressure, were recorded every 15 minutes during the first hour and every 30 minutes thereafter until completion of surgery.

Adverse events were predefined as bradycardia (heart rate <50 beats/min), hypotension (mean arterial pressure <65 mmHg or >20% reduction from baseline), hypertension, arrhythmias, and oxygen desaturation (SpO₂ <92%). All adverse events were recorded and managed according to institutional protocols.

Statistical analysis

All randomized patients completed the study, and no missing data were observed. Data analysis was performed on an intention-to-treat basis. Data were analyzed using SPSS Statistics version 22 (IBM Corp., Armonk, NY, USA).

Continuous variables were expressed as mean ± standard deviation, and categorical variables as frequencies and percentages. Normality was assessed using the Shapiro-Wilk and Kolmogorov-Smirnov tests. Intergroup comparisons were performed using one-way ANOVA or the Kruskal-Wallis test as appropriate. Categorical variables were compared using the chi-square test or Fisher’s exact test. A p-value <0.05 was considered statistically significant. The study was powered for detection of differences in early cognitive outcomes and was not designed to detect rare adverse events.

## Results

A total of 72 patients were enrolled in the study and randomly allocated into three equal groups of 24 each: Group D1, Group D2, and Group P. All patients completed the study. Baseline demographic variables and sex distribution were comparable across the three groups with no statistically significant differences, indicating successful randomization.

Baseline characteristics

Sex distribution did not differ significantly among the three groups (p = 0.944). Other baseline variables, including age, weight, height, body mass index, and ASA physical status, were also comparable across the groups, indicating successful randomization (Table 1).

**Table 1:** Demographic Characteristics: Sex Distribution Across Study Groups Values are expressed as number of patients (%). Intergroup comparison of sex distribution among the three groups was performed using the chi-square test.

Sex	Group D1 (n=24)	Group D2 (n=24)	Group P (n=24)	χ² (df=2)	p-value
Male	15 (62.5%)	14 (58.3%)	14 (58.3%)	0.12	0.944
Female	9 (37.5%)	10 (41.7%)	10 (41.7%)	—	—

Intraoperative hemodynamics

Intraoperative systolic and diastolic blood pressure values remained within clinically acceptable ranges in all three groups. However, significant intergroup differences were observed at several intraoperative time points, beginning from 30 minutes onward, with patients in the dexmedetomidine groups (D1 and D2) demonstrating lower systolic blood pressure values compared with the placebo group (Table 2).

**Table 2:** Mean Systolic Blood Pressure at Different Time Points Values are expressed as mean ± standard deviation. Intergroup comparison at each time point was performed using one-way analysis of variance (ANOVA); corresponding F values and p-values are reported.

Time point	Group D1 (Mean ± SD)	Group D2 (Mean ± SD)	Group P (Mean ± SD)	F (2,69)	p-value
Baseline	123.25 ± 8.84	120.58 ± 7.06	121.50 ± 6.22	0.79	0.457
30 min	120.08 ± 7.97	118.00 ± 9.34	124.08 ± 5.22	3.71	0.029
45 min	115.83 ± 7.95	113.00 ± 12.19	122.00 ± 5.04	6.32	0.003
1 hour	112.08 ± 8.99	110.67 ± 11.18	121.25 ± 5.10	11.24	<0.001

Postoperative cognitive function (MoCA)

At one hour postoperatively, significant differences in MoCA scores were observed among the groups (p = 0.046). The placebo group demonstrated lower early postoperative cognitive scores, particularly in the orientation domain, which showed a statistically significant difference compared with the dexmedetomidine groups (p = 0.001) (Table 3).

**Table 3:** Montreal Cognitive Assessment (MoCA) Component Scores at One Hour Values are expressed as mean ± standard deviation. Intergroup comparison was performed using one-way analysis of variance (ANOVA). Cognitive function was assessed using the Montreal Cognitive Assessment (MoCA) scale [6]. F values and p-values are reported for each domain.

MoCA Domain	Max	Group D1	Group D2	Group P	F (2,69)	p-value
Executive	5	0.04 ± 0.20	0.08 ± 0.28	0.00 ± 0.00	1.03	0.362
Naming	3	2.29 ± 0.69	2.42 ± 0.50	2.04 ± 0.59	1.96	0.146
Attention	6	1.33 ± 0.48	1.13 ± 0.74	1.42 ± 0.78	1.17	0.315
Language	3	0.08 ± 0.28	0.04 ± 0.20	0.00 ± 0.00	1.03	0.362
Abstraction	2	0.04 ± 0.20	0.04 ± 0.20	0.00 ± 0.00	0.5	0.609
Delayed Recall	5	1.50 ± 0.51	1.54 ± 0.66	1.46 ± 0.51	0.13	0.877
Orientation	6	4.46 ± 1.22	4.50 ± 1.14	3.25 ± 1.26	7.42	0.001
Education	1	0.58 ± 0.50	0.71 ± 0.46	0.71 ± 0.46	0.55	0.581
Total MoCA	30	9.38 ± 1.81	9.29 ± 2.29	8.13 ± 1.54	3.18	0.046

MoCA at six hours

By six hours postoperatively, cognitive scores improved in all groups; however, Group D2 demonstrated significantly better cognitive performance across multiple MoCA domains compared with Group D1 and the placebo group (p < 0.001) (Table 4).

**Table 4:** Montreal Cognitive Assessment (MoCA) Component Scores at Six Hours Values are expressed as mean ± standard deviation. Intergroup comparison was performed using one-way analysis of variance (ANOVA). F values and corresponding p-values are shown for each MoCA domain and total score [6].

MoCA Domain	Max	Group D1	Group D2	Group P	F (2,69)	p-value
Executive	5	4.04	4.17	4.46	4.82	0.01
Naming	3	2.75	2.54	2.42	2.71	0.073
Attention	6	3.79	3.67	2.92	14.56	<0.001
Language	3	1.79	1.96	1.54	3.3	0.044
Abstraction	2	1.25	1.46	1.13	3.79	0.027
Delayed Recall	5	3.17	3.88	2.88	15.21	<0.001
Orientation	6	5.5	5.46	5.04	7.39	0.001
Total MoCA	30	23.04 ± 2.03	24.04 ± 1.65	21.21 ± 1.18	22.84	<0.001

Clinically significant cognitive decline

A decrease of more than five points from baseline on the MoCA scale was considered clinically significant postoperative cognitive decline. The placebo group demonstrated the highest incidence of such decline (37.5%), whereas both dexmedetomidine groups showed a lower incidence of 12.5% each (Table 5).

**Table 5:** Distribution of Patients with Clinically Significant Montreal Cognitive Assessment (MoCA) Decline (>5 Points) Values are expressed as number of patients. Intergroup comparison was performed using the chi-square test. Clinically significant cognitive decline was defined as a decrease of more than five points on the MoCA scale [6].

Group	MoCA difference <5	MoCA difference >5	χ² (df=2)	p-value
D1	21	3		
D2	21	3	6	0.048
P	15	9		

## Discussion

The present randomized controlled study evaluated the effect of two different intraoperative dexmedetomidine dosing regimens on early postoperative cognitive decline in elderly patients undergoing major abdominal surgery under general anaesthesia. Our findings demonstrate that dexmedetomidine, administered either as a moderate (D1) or higher (D2) dose, significantly preserved early postoperative cognitive function compared with placebo, with the higher dose providing the greatest neurocognitive benefit. These results support growing evidence that dexmedetomidine exerts a dose-dependent protective effect on postoperative cognition in older adults exposed to major surgical stress [8].

In our study, patients in the placebo group exhibited significantly lower MoCA scores at one hour and six hours postoperatively and a higher proportion of clinically meaningful cognitive decline (>5-point drop). This pattern is consistent with previous reports showing that transient early postoperative cognitive impairment is common in elderly individuals following major abdominal surgery due to heightened neuroinflammatory responses and perioperative physiological stress [9]. The observed early decline highlights the importance of perioperative modulation of inflammatory and sympathetic pathways in mitigating cognitive vulnerability in this population.

Dexmedetomidine’s preservation of cognitive function likely relates to its anti-inflammatory and neuroprotective properties. Previous studies have demonstrated reductions in pro-inflammatory cytokines such as IL-6 and TNF-α, along with improved cerebral perfusion through sympathetic attenuation [10,11]. While our study did not include biomarker assessment, the observed stabilization of intraoperative hemodynamics and improved MoCA scores in the dexmedetomidine groups indirectly support these proposed mechanisms.

The higher-dose dexmedetomidine group (D2) demonstrated superior cognitive scores at six and 24 hours, suggesting a dose-response relationship. Similar findings have been reported in prior dose-comparison studies, where higher intraoperative dexmedetomidine doses were associated with improved early cognitive trajectories without a significant increase in adverse events [12]. Although bradycardia and hypotension remain known dose-related risks, no clinically significant differences in adverse events were observed within the dosing range used.

Importantly, although early cognitive decline was evident in the placebo group, partial recovery by 24 hours suggests that the observed impairment likely reflects early POCD rather than established long-term POCD, which is traditionally assessed days to months after surgery [13-15]. Therefore, our findings should be interpreted as reflecting early postoperative cognitive outcomes rather than definitive long-term neurocognitive prognosis. Also, the results should not be extrapolated to long-term POCD.

Limitations 

This study has several limitations that should be considered. First, cognitive assessment was limited to the first 24 hours postoperatively; therefore, conclusions regarding persistent or long-term POCD cannot be drawn. The findings primarily reflect early POCD, which may be transient and mechanistically distinct from delayed or persistent POCD.

Second, biochemical markers of neuroinflammation or neuronal injury (e.g., IL-6, TNF-α, NSE, S100β) were not measured, limiting direct mechanistic correlation between dexmedetomidine administration and neuroprotective effects.

Third, although a standardized anaesthetic technique was used, perioperative variables that may influence cognition, such as depth of anaesthesia, intraoperative fluid management, postoperative sedation exposure, and analgesic variability, were not fully controlled or quantified, introducing potential confounding effects.

Fourth, inclusion was restricted to ASA physical status I-II patients, which limits the generalizability of the findings to higher-risk elderly populations with significant comorbidities.

Finally, although the study was adequately powered for detecting differences in cognitive outcomes, the modest sample size may be insufficient to identify rare adverse events associated with higher dexmedetomidine doses.

Future directions

Future research should focus on longer follow-up durations, including cognitive assessment at one week, one month, and three months postoperatively, to determine whether intraoperative dexmedetomidine confers sustained cognitive protection. Incorporating inflammatory and neuronal biomarkers would help clarify the mechanistic pathways underlying dexmedetomidine’s neuroprotective effects. Larger multicenter randomized trials involving elderly patients across varied risk profiles, including ASA III and IV categories, are needed to improve external validity. Additionally, comparative studies evaluating dexmedetomidine against other emerging neuroprotective agents or multimodal strategies could further refine perioperative cognitive protection protocols. Exploring individualized dosing strategies based on age-related pharmacodynamic changes may also help optimize safety and efficacy in geriatric populations.

## Conclusions

In this randomized controlled study of elderly patients undergoing major abdominal surgery under general anaesthesia, intraoperative dexmedetomidine was associated with a significant reduction in early postoperative cognitive decline within the first 24 hours after surgery. Both dexmedetomidine dosing regimens resulted in higher postoperative MoCA scores compared with placebo, with the higher dose demonstrating the greatest preservation of early cognitive function. The lower incidence of clinically significant cognitive decline (>5-point decrease in MoCA score) further supports the beneficial effect of dexmedetomidine on early postoperative cognitive outcomes.

These findings suggest that dexmedetomidine, particularly at higher intraoperative doses, may be safely incorporated into anaesthetic protocols to improve early postoperative cognitive recovery in elderly surgical patients. While the observed benefits may relate to hemodynamic stabilization and attenuation of perioperative stress responses, definitive neuroprotective mechanisms cannot be confirmed in the absence of biomarker assessment. Longer-term follow-up and larger multicenter studies are required to determine whether these early cognitive improvements translate into sustained postoperative neurocognitive benefit.
